# *Arabidopsis *gene co-expression network and its functional modules

**DOI:** 10.1186/1471-2105-10-346

**Published:** 2009-10-21

**Authors:** Linyong Mao, John L Van Hemert, Sudhansu Dash, Julie A Dickerson

**Affiliations:** 1 Virtual Reality Applications Center, Iowa State University, Ames, IA 50010, USA; 2 Program of Bioinformatics and Computational Biology, Iowa State University, Ames, IA 50010, USA; 3 Department of Electrical and Computer Engineering, Iowa State University, Ames, IA 50010, USA

## Abstract

**Background:**

Biological networks characterize the interactions of biomolecules at a systems-level. One important property of biological networks is the modular structure, in which nodes are densely connected with each other, but between which there are only sparse connections. In this report, we attempted to find the relationship between the network topology and formation of modular structure by comparing gene co-expression networks with random networks. The organization of gene functional modules was also investigated.

**Results:**

We constructed a genome-wide *Arabidopsis *gene co-expression network (AGCN) by using 1094 microarrays. We then analyzed the topological properties of AGCN and partitioned the network into modules by using an efficient graph clustering algorithm. In the AGCN, 382 hub genes formed a clique, and they were densely connected only to a small subset of the network. At the module level, the network clustering results provide a systems-level understanding of the gene modules that coordinate multiple biological processes to carry out specific biological functions. For instance, the photosynthesis module in AGCN involves a very large number (> 1000) of genes which participate in various biological processes including photosynthesis, electron transport, pigment metabolism, chloroplast organization and biogenesis, cofactor metabolism, protein biosynthesis, and vitamin metabolism. The cell cycle module orchestrated the coordinated expression of hundreds of genes involved in cell cycle, DNA metabolism, and cytoskeleton organization and biogenesis. We also compared the AGCN constructed in this study with a graphical Gaussian model (GGM) based *Arabidopsis *gene network. The photosynthesis, protein biosynthesis, and cell cycle modules identified from the GGM network had much smaller module sizes compared with the modules found in the AGCN, respectively.

**Conclusion:**

This study reveals new insight into the topological properties of biological networks. The preferential hub-hub connections might be necessary for the formation of modular structure in gene co-expression networks. The study also reveals new insight into the organization of gene functional modules.

## Background

Biological networks characterize the interactions of biomolecules at a systems-level and can help us better understand how biomolecules interact with each other to carry out biological functions in living cells. In the representation of biological networks, it is natural to use graph to describe the interactions between biomolecules. A node in a graph represents a biomolecule such as a gene, a protein or a metabolite, and an edge (or link) indicates the interaction between these two biomolecules. Such interactions could be physical interactions, metabolite flow, regulatory relationships, co-expression relationships, *etc*. [1]. One important property of networks is the modular structure, in which nodes are densely connected with each other, but between which there are only sparse connections [2]. Biomolecules belonging to the same module interact with each other to carry out a specific biological function.

The rapid accumulation of genome-wide gene expression data allows the creation of gene co-expression networks by examining the co-expression patterns of genes over a large number of experimental conditions. In the gene co-expression network, a node is a gene, and an edge is drawn between gene *A *and *B *if the correlation coefficient between these two genes is above a threshold. Gene co-expression networks have proven useful in analyzing microarray data in model organisms including yeast, mouse and human [3-10]. In plants, since the complete sequencing of the *Arabidopsis thaliana *genome in 2000, thousands of microarray experiments under diverse conditions have been conducted, and the array data have been deposited in public databases. Accordingly, genome-wide *Arabidopsis *gene co-expression networks (AGCNs) have also been constructed by calculating the pairwise gene expression correlations over a large number of microarray experiments, ranging from over 300 arrays to more than 2000 arrays [11-16].

In detecting gene functional modules (or clusters) from gene co-expression networks, a guide-gene approach is commonly used. In this approach, a set of genes with known functions, termed as guide genes (or bait genes), were used to query the gene co-expression network. A subnetwork comprising of the guide genes and the genes that were connected to the guide genes within a user-defined distance was retrieved. A gene module was then considered to be equivalent of the retrieved subnetwork itself [13], or it was extracted from the subnetwork using visualization tools [11,14] or maximal-clique-finding method [12]. Using the guide-gene approach, one can find gene modules that are associated with a specific biological function or metabolic process [11,13]. However, the drawback of this approach is that a module found in this way might be incomplete and belong to a larger and more densely connected module [11]. In addition, using visualization to extract modules is subjective and affected by users' judgments. To avoid such drawbacks, an alternative approach, top-down approach (or non-targeted approach), is used to naturally partition the network into modules by applying graph clustering algorithms. Compared with the guide-gene approach that requires the prior knowledge about the seed genes, the top-down approach is relatively knowledge independent and novel hypotheses might be developed from the clustering result [4,6,16].

In this report, we used a top-down approach to identify and evaluate gene functional modules from large *Arabidopsis *microarray data sets. First, we constructed AGCN by using more than 1000 high quality microarrays. Then, we analyzed the topological properties of the network and extracted modules from the network by using Markov Clustering (MCL) Algorithm [17]. The functional coherence of the extracted modules was evaluated. In this report, we attempted to assess if there exists intrinsic modular structure in AGCN and find the relationship between the network topology and formation of modular structure by comparing the real biological network with random networks. We then focused our analysis on two gene functional modules, photosynthesis module and cell cycle module, that are central to plant growth and development. A close examination of the organization of these two modules reveals that both modules involve multiple biological processes coordinated at the transcriptional level. Although our findings are based on the analysis of *Arabidopsis *microarray data, the uncovered network properties and organization of gene functional modules may have implications in non-plant organisms and other types of biological networks such as protein interaction networks.

## Results

**Figure 1:**
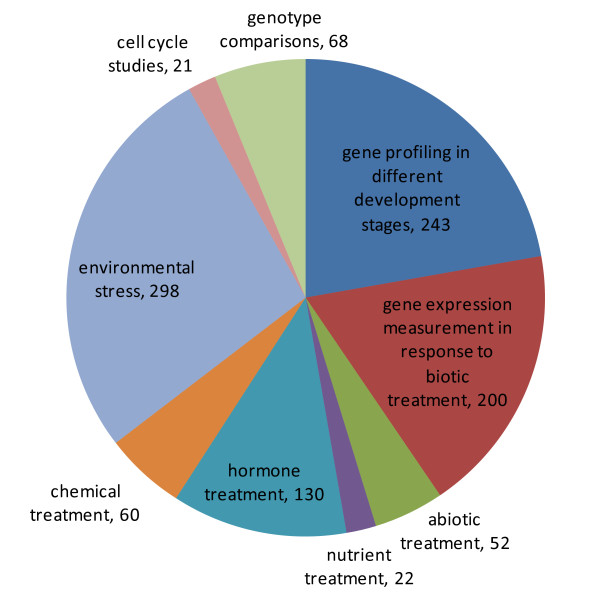
**Composition of the 1094 ATH1 arrays according to the experimental conditions they represent**. For a detail description of the arrays, refer to the TAIR web site .

We used 1094 non-redundant Affymetrix ATH1 arrays from the AtGenExpress consortium to calculate the pairwise correlations between genes. These arrays were normalized to the same scale by employing MAS algorithm (see Methods). The AtGenExpress array data has been shown to be highly reliable and reproducible [18,19]. Figure 1 shows the experimental conditions of these 1094 ATH1 arrays. The high quality of the microarray data and diverse experimental conditions allow us to capture the true co-expression relationship between two genes.

To circumvent the correlation computed from noise, we used only the genes that showed significant changes across the 1094 conditions (see Methods). Of the 22746 *Arabidopsis *probe sets on the ATH1 chip, 16293 (72%) were selected. Next, the genes' expression values were log transformed and Pearson correlation coefficient (Pcc) was computed between each pair of the 16293 genes (see Methods).

**Figure 2:**
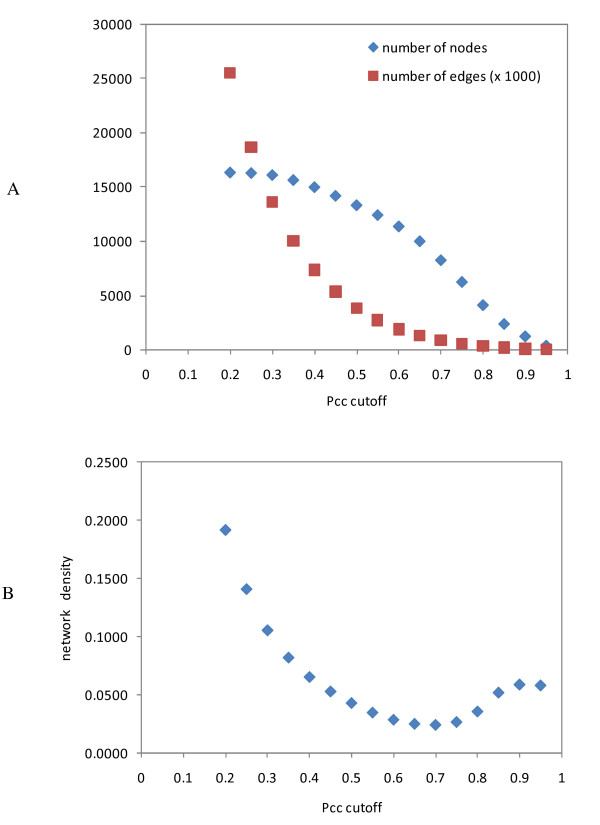
**Choosing Pcc cutoff values**. (A) The number of nodes and number of edges as a function of Pcc cutoff value. Only edges with Pcc greater than the cutoff value were used to construct the co-expression network. Only nodes connected by these edges were used in our network analysis. (B) Network densities at different Pcc cutoff values.

To choose an appropriate Pcc cutoff value, we examined the changes in the node number, edge number, and network density as a function of Pcc cutoff values. As the cutoff value increased, both the node number and edge number decreased (Figure 2A); however, as the cutoff reached a relatively high value, the decreasing rate of edges became slower than that of nodes, which might lead to an increase in the network density. Indeed, as shown in Figure 2B, the network density showed minima around 0.70 Pcc cutoff value and increased thereafter. A Pcc cutoff value greater than 0.70 would be appropriate so that edges with high Pcc values would densely connect a decreasing number of nodes, which would facilitate the following detection of biologically meaningful modules [11]. In this study, Pcc cutoff value was set to 0.75 so that a relatively large number of nodes could be retained in the network. At this relatively stringent cutoff value, only the top 0.39% of all possible edges among the 16293 genes with respect to their Pcc values were retained. The resulting AGCN contains 6206 nodes, 512,936 edges, and a network density of 0.0266. For comparison, three random networks that each preserved the node numbers and node degrees of AGCN were also created (see Methods).

### Network Topology

**Figure 3:**
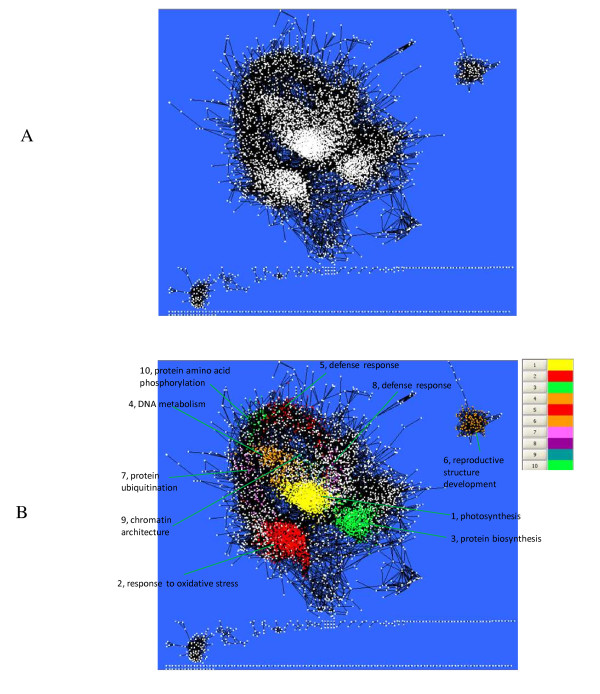
**Network topology displayed using the yFiles Organic Layout algorithm in Cytoscape **[20]. (A) Layout of the *Arabidopsis *gene co-expression network. A white rectangle represents a node (*i.e*. probe set). A black edge connecting two nodes indicates the co-expression relationship between these two nodes. (B) Mapping the 10 largest modules onto the network. The most over-represented biological process GO term was also shown with each module.

Figure 3A displays a layout of AGCN using the Cytoscape software package [20]. The network comprises of 100 disconnected components. Within each component, each pair of nodes was directly or indirectly connected. The major component in the network has 5743 (92.5%) nodes. The smallest component contains only two nodes, and 68 such components were found. The qualitative global topology of the AGCN is similar to that of the yeast protein interaction network which comprises of a major component covering 93% of the network nodes and many small components [21].

**Figure 4:**
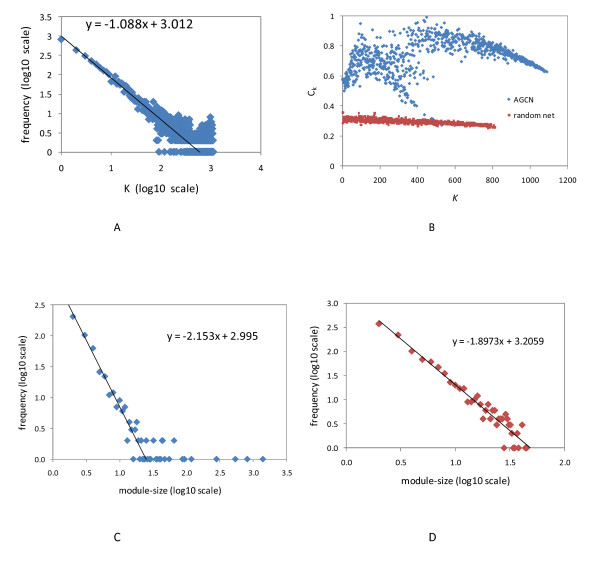
**Topological properties of the *Arabidopsis *gene co-expresion network (AGCN) and the GGM network **[13]. (A) Distribution of the node degree (*K*) for AGCN. (B) Comparing the distribution of the clustering coefficient (*C*_*k*_) with respect to the node degree between AGCN and random networks. The three random networks exhibited almost identical distributions. For clarity, only one random network's distribution was shown. (C) The distribution of the module size for AGCN. (D) The distribution of the module size for GGM network.

On average, each node in AGCN has 165 co-expression links, but the distribution of the node degrees is highly skewed. The distribution fits to a power law distribution with a tail (Figure 4A), indicating that the network is scale free. Interestingly, we found that the top 382 nodes (genes) in terms of their degrees connected to each other and formed a 382-member clique. Each of these 382 genes has at least 889 co-expression links. We then examined the immediate neighbors of these 382 genes. Surprisingly, these 382 genes were connected to only 1099 other genes in AGCN, whereas the same set of genes were linked to 4913, 4964 and 4970 other genes in the three random networks, respectively. Thus, unlike the random network, these hub genes did not reach out to the entire *Arabidopsis *gene co-expression network. They were rather densely connected only to a fraction of the network. Since a module is a subnetwork which is densely connected within itself but sparsely connected with rest of the network, these 382 genes and many of their densely connected neighbors will form a large module with the clique structure serving as the module's core. It was later confirmed by network clustering results (see below).

We analyzed the functions of the 382 hub genes using gene ontology (GO) (see Methods). Of the 382 genes, 335 were annotated with cellular component GO terms. Interestingly, among the 335 genes, the products of 265 genes (79%) were located in chloroplast with a p-value as low as 1.87E-132. Table 1 lists the biological process GO terms that were significantly over-represented in the hub genes. The most dominant term was photosynthesis with respect to p-values. The 382 genes forming a co-expression clique exhibited a maximal degree of coordination at the transcriptional level, suggesting that these genes might be involved in a common biological function. Based on the gene ontology analysis and the observation that several over-represented biological processes (*e.g*. electron transport, pigment metabolism, glucose metabolism) are coupled with photosynthesis, the 382 genes are very likely to function in photosynthesis. Additional evidence towards this conclusion is provided below.

**Table 1:** Comparing significantly enriched biological process GO terms in the 382 hub genes with module 1.

**GO term**	**Hub-genes** **p-value^1^**	**Module 1** **p-value**
cell redox homeostasis	4.99E-05	NA

chloroplast organization and biogenesis	NA	7.01E-08

cofactor metabolism	1.86E-06	1.93E-18

electron transport	5.49E-06	1.80E-06

glucose metabolism	7.61E-05	NA

glycine catabolism	7.92E-05	NA

photosynthesis	1.38E-24	1.38E-52

pigment metabolism	3.48E-07	3.03E-12

protein biosynthesis	1.15E-07	3.05E-07

protein folding	5.70E-05	NA

vitamin metabolism	NA	8.41E-06

1. 'NA' indicates the GO term was not significantly over-represented.

To further evaluate the network topology, we analyzed the property of clustering coefficients. The clustering coefficient of a given node, *C*_*n*_, measures how close the node *n *and its directly connected neighbors resemble a clique (see Methods). The AGCN exhibited an average clustering coefficient, <*C*_*n*_>, of 0.640, whereas the three random networks exhibited an average clustering coefficient of 0.304, 0.305 and 0.306, respectively. That <*C*_*n*_> of AGCN is more than twice of the random network indicated the potential modularity in the co-expression network [22]. The distribution of the clustering coefficient (*C*_*k*_) with respect to the node degree (*k*) further distinguished the co-expression network from the random networks (Figure 4B). For each random networks, *C*_*k *_was approximately a constant with respect to *k*, and the variation of *C*_*k *_was small (stdev = {0.0156, 0.0162, 0.0164}). For the AGCN, *C*_*k *_exhibited a complex relationship with *k*, and the variation of *Ck *was much larger (stdev = 0.107). The complex relationship between *C*_*k *_and the node degree may affect how modules are organized in AGCN [22].

### Network Clustering Analysis

We used the MCL algorithm to partition AGCN into gene modules. MCL is an efficient graph clustering algorithm based on the simulation of random walks within a graph. MCL has been applied to detect modules in yeast protein interaction networks [23] and protein family networks [24]. A recent study, which evaluated four clustering algorithms for protein interaction networks, showed the superior performance of MCL in the identification of protein complexes [25]. The algorithm is very efficient and took only two minutes to perform clustering on the AGCN (the Linux command: *mcl AT-cor-net-0d75 -I 1.8 -- abc -scheme 7*) on a 3.6 GHz Intel Xeon CPU with 3 GB memory.

**Figure 5:**
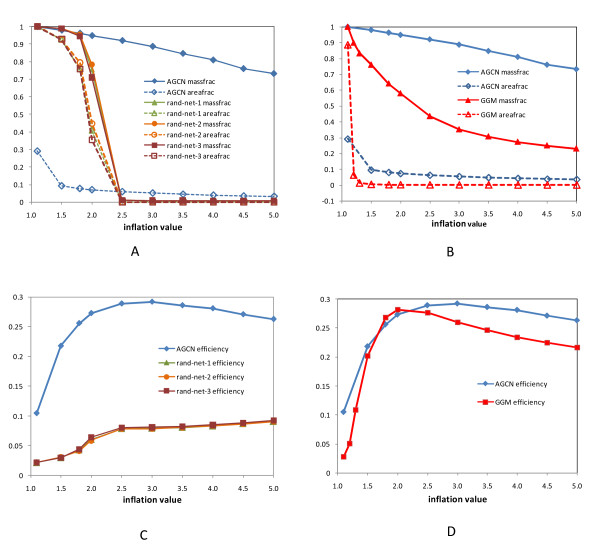
**Assessment of the quality of network clustering**. (A) Comparing the effects of inflation values on area fraction and mass fraction between AGCN and three random networks. Clustering on a network with the intrinsic modular structure should produce a small area fraction but a large mass fraction close to one. This is indeed the case for the AGCN. In contrast, all three random networks had to use a large area fraction to capture a large mass fraction, suggesting the absence of modular structure. (B) Comparing the effects of inflation values on area fraction and mass fraction between AGCN and GGM network. (C) Comparing the effects of inflation values on efficiency between AGCN and three random networks. The efficiency aims to balance between the objective to obtain a high mass fraction and the objective to keep the area fraction low. A higher efficiency indicates a better performance on network clustering by using some mathematical criteria. A formal definition of efficiency can be found in [64]. (D) Comparing the effects of inflation values on efficiency between AGCN and GGM network.

The MCL algorithm has an important parameter, the Inflation parameter (*I*). A higher value for *I *tends to produce a larger number of modules with a smaller module size. We tested different inflation values on the AGCN as well as the three random networks. We used area fraction, mass fraction and efficiency (see Methods) to assess the overall quality of the network clustering. Since a module is a densely connected subnetwork and the connections between modules are sparse, clustering on a network with the intrinsic modular structure should produce a small area fraction but a large mass fraction close to one. This is indeed the case for the AGCN (Figure 5A). For example, when *I *was set to 1.5, clustering on AGCN captured 97.9% of the entire edge masses by using only 9.3% of the network area, reflecting the presence of modular structure in AGCN. In contrast, with the same inflation value, all three random networks had to use 93% of the network area to capture a similar mass fraction (Figure 5A), suggesting the absence of modular structure. An appropriate value for *I *is between 1.5 and 3.0. Within this range, clustering on the co-expression network used 5 - 10% of the area to capture more than 85% of the entire edge masses. The above analysis is purely mathematical. Recently Brohee and van Helden evaluated the MCL algorithm in identifying protein complexes from yeast protein interaction networks, and they chose 1.8 as the optimal value for *I *based on the analysis of 42 artificial biological networks that simulated the data sets obtained from high-throughput experiments [25]. They also found that when *I *was set at 1.8, MCL was resilient to network noise.

With the inflation value set to 1.8, MCL detected 527 modules from the AGCN. The ten largest modules were mapped to the network (Figure 3B). Similar to the node degree distribution, the module size distribution is also highly skewed. The largest module had 1381 nodes whereas 86% of the modules had fewer than 10 nodes. The average size of the modules is 11.8 and the median is 3. The log-log plot of frequency versus module size demonstrates that the distribution of the module size followed a power law distribution with tails (Figure 4C). With the same parameter setting, the largest module extracted from the three random networks contained 5398, 5403 and 5528 nodes, respectively. And the size of the second largest module only ranged from 9 to 13 nodes. The module size distribution further indicates the lack of modular structure in the random network.

### Module Annotation

**Figure 6:**
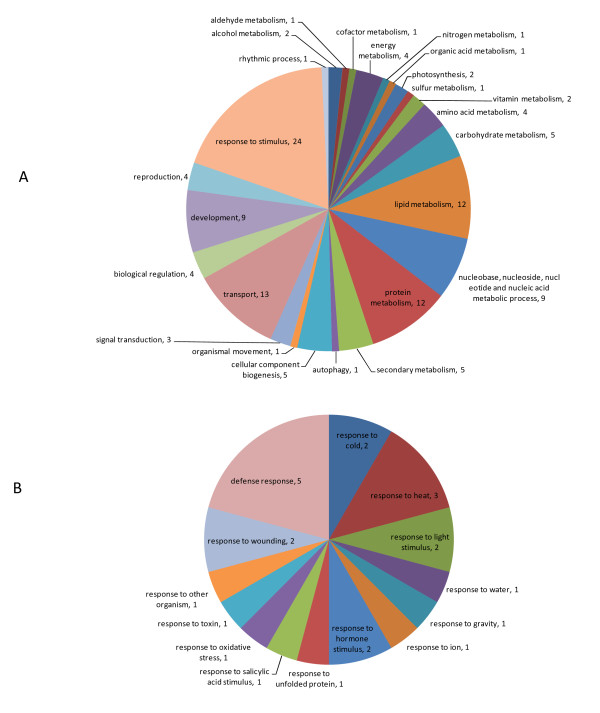
**(A) Functional annotations of 127 modules with significantly over-represented biological process GO terms**. The number associated with each annotation indicates the number of modules annotated to that category. See our web site for the list of 127 modules. (B) Composition of the 24 modules that were annotated to response to stimulus.

Since genes belonging to the same module are co-expressed across diverse conditions, functional coherence among the module members is expected. We carried the enrichment analysis of biological process GO terms in 317 modules containing 3 or more members. 127 of the 317 modules (40%) had GO terms that were significantly over-represented (*i.e*. FWER-adjusted p-value < 0.05, see Methods). We categorized these 127 modules by manual annotations (Figure 6A). Not surprisingly, the largest group is associated with 'response to stimulus' (Figure 6B), the major theme of microarray experiments in AtGenExpress (Figure 1).

**Figure 7:**
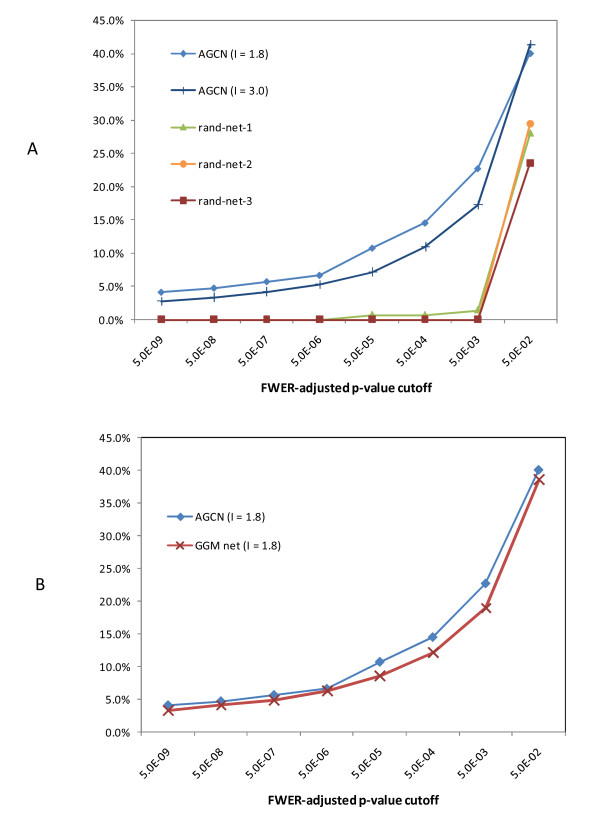
**Percentage of modules with three or more members that had significantly over-represented biological process GO terms using different p-value cutoffs**. For example, the data points at p-value cutoff of 5.0E-02 indicate the percentage of modules that had enriched GO terms with Bonferroni Family-Wise Error Rate (FWER) adjusted p-values less than 5.0E-02. (A) Comparing AGCN with three random networks. (B) Comparing AGCN with GGM network.

Furthermore, we observed that 46 of the 317 modules (14.5%) in AGCN had over-represented GO terms with FWER-adjusted p-values below 5E-4; whereas all three random networks had less than 1% of the modules with over-represented GO terms at such significance level (Figure 7A). The annotations for these 46 modules include both central metabolic processes and specific cellular functions (Table 2). In addition, we compared the clustering result for AGCN using 1.8 inflation value with that using 3.0. Although MCL clustering showed maximal efficiency at 3.0 (Figure 5C), the clustering result at 1.8 inflation value produced a higher percentage of functionally coherent modules (Figure 7A).

**Table 2:** Significantly enriched GO terms in AGCN modules (*I *= 1.8)

**module**	**# annotated genes^1^**	**GO term^2^**	**genes in GO term^3^**	**p value^4^**
1	1208	photosynthesis	97/107	1.38E-52

2	684	response to oxidative stress	30/88	1.43E-07

3	438	protein biosynthesis	95/204	5.68E-52

4	256	DNA metabolism	59/107	9.07E-52

5	104	defense response	18/205	4.79E-08

6	87	reproductive structure development	15/100	3.16E-11

7	78	protein ubiquitination	5/14	1.00E-06

8	59	defense response	19/205	1.65E-13

9	48	establishment and/or maintenance of chromatin architecture	6/43	1.71E-06

11	39	cell wall modification	5/32 2	64E-06

15	36	response to wounding	7/54	4.50E-08

18	33	cuticle biosynthesis	5/8	3.45E-10

21	29	toxin metabolism	5/25	1.54E-07

27	23	glucosinolate biosynthesis	4/9	3.12E-08

28	18	RNA processing	5/65	1.65E-06

31	20	monosaccharide metabolism	5/47	5.67E-07

33	17	response to heat	10/47	1.69E-17

34	18	secondary cell wall biosynthesis (sensu Magnoliophyta)	6/8	1.51E-14

35	17	protein biosynthesis	11/204	1.75E-12

41	16	enzyme linked receptor protein signaling pathway	5/51	2.49E-07

42	15	organic acid metabolism	9/186	2.38E-10

43	13	response to auxin stimulus	7/69	6.62E-11

46	14	lipid metabolism	7/194	1.93E-07

50	12	cellular respiration	6/15	1.34E-13

56	11	leaf development	4/28	1.87E-07

67	10	starch metabolism	8/20	3.18E-19

79	9	indoleacetic acid metabolism	3/4	1.28E-08

80	8	phenylpropanoid metabolism	6/44	5.71E-12

119	6	response to heat	6/47	3.14E-13

122	6	lipid transport	4/47	7.49E-08

140	5	nitrogen compound metabolism	5/129	7.23E-09

154	4	wax biosynthesis	2/7	8.65E-06

165	4	glutamate biosynthesis	2/2 4	12E-07

170	4	electron transport	4/219	2.64E-06

173	4	fatty acid beta-oxidation	2/9	1.48E-05

180	4	purine transport	2/3	1.24E-06

190	4	response to water deprivation	4/41	2.87E-09

201	2	Glycolysis	2/16	8.24E-06

205	3	RNA splicing, via transesterification reactions with bulged adenosine as nucleophile	2/5	2.06E-06

213	4	response to heat	4/47	5.05E-09

224	3	fatty acid beta-oxidation	2/9	7.41E-06

238	3	sulfolipid biosynthesis	2/2	2.06E-07

266	3	ovule development	2/12	1.36E-05

273	3	phenylpropanoid metabolism	3/44	5.06E-07

311	3	Proteolysis	3/130	1.37E-05

315	3	response to iron ion	2/3	6.18E-07

1. The number of genes which were assigned with biological process GO terms in a module.

2. Only the most over-represented GO term was listed for each module.

3. The two values listed in this column refer to the number of genes associated with the over-represented GO term in the module and the number of genes associated with the same GO term in the network.

4. The p value indicated the probability that a module contains equal or larger number of genes associated with the GO term under a hypergeometric distribution.

In addition to analyzing the enrichment of GO terms in modules, we also analyzed the over-representation of pathway terms (see Methods). However, only 10% of the 6206 genes in AGCN have been annotated as metabolic pathway genes. Among the 39 modules, each of which contained at least three annotated pathway genes, 26 were detected with significantly enriched pathway terms. Ten of them (FWER-adjusted p-value < 5E-4) were listed in table 3. The table also indicates the correspondence between the enriched pathway terms and biological process GO terms.

**Table 3:** Significantly enriched pathway terms in AGCN modules (*I *= 1.8).

**module**	**# pathway genes^1^**	**Pathway term^2^**	**GO term^3^**	**genes in pathway term^4^**	**p value**
1	192	photosynthesis, light reaction	Photosynthesis	27/27	4.69E-15

3	22	*de novo *biosynthesis of purine nucleotides	purine nucleoside monophosphate biosynthesis	7/10	2.84E-09

4	5	de novo biosynthesis of pyrimidine deoxyribonucleotides	DNA metabolism	3/6	5.02E-06

11	11	homogalacturonan degradation	cell wall modification	10/34	6.47E-13

45	6	acetyl-CoA biosynthesis (from citrate)	acetyl-CoA biosynthesis	2/2	7.82E-05

67	6	starch degradation	starch metabolism	5/11	3.66E-09

79	8	glucosinolate biosynthesis from tryptophan	indoleacetic acid metabolism	5/5	7.45E-11

80	6	flavonoid biosynthesis	phenylpropanoid metabolism	4/6	3.67E-08

140	3	tryptophan biosynthesis	nitrogen compound metabolism	3/5	2.53E-07

273	3	salicylic acid biosynthesis	aromatic compound biosynthesis	2/3	4.69E-05

1. The number of genes which were annotated as pathways genes in a module.

2. Only the most over-represented pathway term was listed for each module.

3. The column lists the over-represented GO term that matches or relates to the pathway term for each module.

4. The two values listed in this column refer to the number of genes annotated to the over-represented pathway in the module and the number of genes annotated to the same pathway in the network.

The effectiveness of our approach is best illustrated by the correspondence of these computational modules with actual biological entities. Three of these modules are examined in detail from this perspective and are presented below.

### Module 1 - Photosynthesis

Module 1, the largest module in AGCN, had 1381 nodes, 399922 edges and a density of 0.42. As shown previously, the 382 hub genes in AGCN formed a clique and altogether connected to 1099 neighbors. All of the 382 hub genes and 927 of their neighbors were included in module 1 and constituted 95% of the module members.

**Figure 8:**
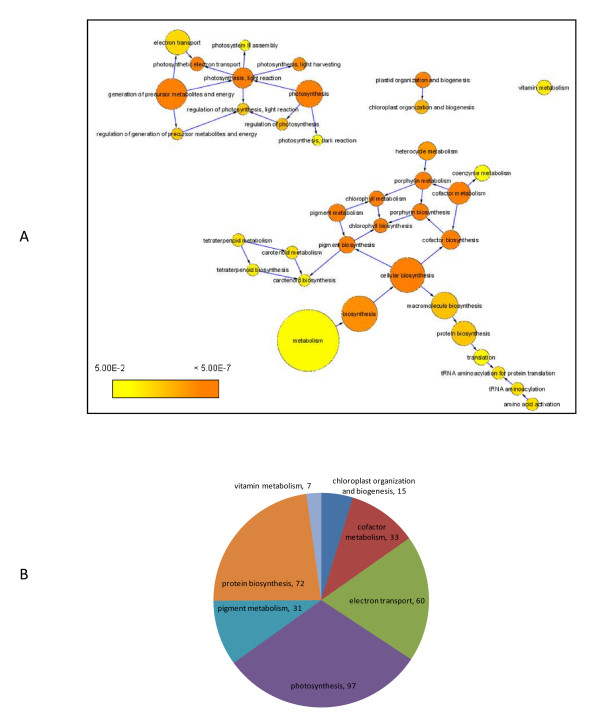
**Functional analysis of module 1**. (A) Significantly over-represented biological process GO terms detected in module 1. Each colored circle represents an over-represented GO term. The color scale indicates the p value of the over-represented GO term. An arrow from GO term A to Go term B indicates that A is the parent of B. (B). Seven major biological process GO terms retrieved from (A). The number following each major GO term refers to the number of genes that were annotated to that category. See our web site for the gene lists.

The significantly over-represented biological process GO terms detected in module 1 are depicted in Figure 8A. They were consolidated into seven major GO terms based on their hierarchical relations (Figure 8B, see methods). Five of the seven major GO terms are also significantly over-represented in the 382 hub genes (Table 1). The p-values for protein biosynthesis and electron transport GO terms are similar between module 1 and the hub genes, respectively. However, the p-values for the other three GO terms (photosynthesis, cofactor metabolism, and pigment metabolism) in module 1 are much more significant than those in the hub genes (Table 1), indicating that module 1 was formed by recruiting more functionally related genes to the 382-member clique.

Among the seven major GO terms, photosynthesis is the most over-represented biological process in module 1 (Table 1), and three other processes (electron transport, pigment metabolism, and chloroplast organization and biogenesis) bear direct physiological connections/associations with photosynthesis. We show below that the remaining three major processes (cofactor metabolism, protein biosynthesis, and vitamin metabolism) are also strongly integrated with photosynthesis, respectively. Thus, photosynthesis becomes the uncontested umbrella process for module 1.

The 33 cofactor metabolism genes in module 1 included 6 genes involved in ATP biosynthesis, 7 genes in NADPH regeneration, and 12 genes in the biosynthesis of photosynthetic electron carriers such as Fe-S cluster, quinones and hemes.

Among the 72 protein biosynthesis genes, the potential locations of the products of 63 genes could be assigned to chloroplast based on their cellular component GO terms, genes' annotations [26], and literatures (see Additional file 1 - Table S1). These 63 genes included 41 genes which might function as the structural constituents of the chloroplast ribosome, 8 genes involved in translation initiation/elongation/release, and 7 genes involved in tRNA aminoacylation. These protein biosynthesis genes are probably involved in the synthesis of photosystem proteins inside chloroplast. For example, *RPS17 *and *RPL9 *encode two chloroplast ribosomal proteins, and the transcripts of these two genes were much more abundant in leaves and stems than they were in roots [27]. In a mutation of *Arabidopsis *where the *RPS17 *expression was dramatically reduced, the activity of the photosystem I (PSI) was significantly reduced [28]. The *HCF107 *gene encodes a protein localized to the chloroplast membrane [29]. The experimental results demonstrated the critical role of HCF107 in the 5'-end processing/stability and/or translation of the *psbH *(*photosystem II protein H*) gene as well as in the translation of the *psbB *gene [29]. *HCF109 *encodes a peptide chain release factor 2, which is involved in the process of translational termination in chloroplasts [30]. In the *HCF109 *mutant, the protein abundances for two ATP synthase subunits, the photosystem I PsaC, the photosystem II PsbB, and PsbZ were substantially reduced [30].

The seven vitamin metabolism genes are involved in vitamin B1 (2 genes), B2 (1 gene), B6 (1 gene), C (2 genes), and E (1 gene) biosynthesis. Among the seven genes, *AT5G28840 *and *VTC2 *encode enzymes involved in the Ascorbic acid (AsA, vitamin C) biosynthesis pathway. It was shown that the light regulation of AsA biosynthesis in *Arabidopsis *leaves is dependent on the photosynthetic electron transport chain [31]. On the other hand, AsA is a potent antioxidant which could detoxify the reactive oxygen generated by photosynthesis and adverse environmental conditions [32]. Ascorbate-deficient mutant of *Arabidopsis *exhibited the symptoms of chronic photooxidative stress when grown in high light [33]. Other vitamins such as vitamin B6 and E could also function as potent antioxidants and protect plants from the photooxidative stress [34,35].

With respect to over-represented pathway terms in module 1, both light reaction and dark reaction of photosynthesis were significantly over-represented (see Additional file 1 - Table S2). The pathways to synthesize two photosynthetic pigments, chlorophyll and carotenoid, were also over-represented. When we looked at cellular component GO terms, the products of 59% of 1148 annotated genes in module 1 could be assigned to the chloroplast (p-value = 4.9E-265).

**Figure 9:**
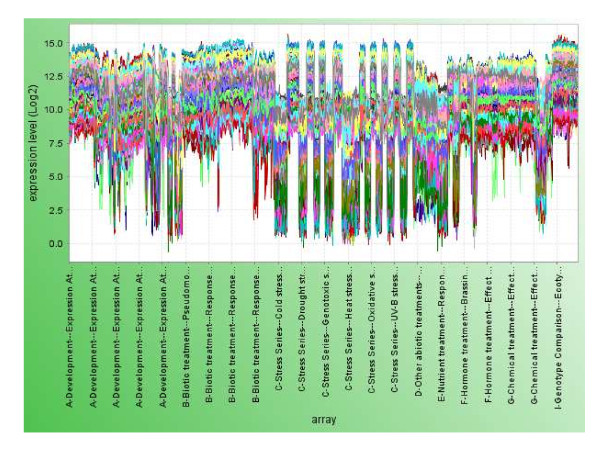
**Tight co-expression of the 382 hub genes across all 1094 arrays in AtGenExpree**. The figure was generated using MetaOmGraph, a component of the MetNet bioinformatics platform [67].

Following the functional analysis of genes in module 1, we examined their transcriptional activities under different conditions. Here, we focused our examination on the 382 hub genes which formed a clique and served as the core of the module. The gene expression behavior of the hub genes should be a typical representation of module 1. Overall, the hub genes exhibited a tightly controlled co-expression pattern across the 1094 conditions profiled in AtGenExpress (Figure 9). Particularly, these hub genes showed an oscillatory expression pattern over the 272 conditions which consisted of nine environmental stresses [18] (see Additional file 1 - Figure S1A). The average expression level for each hub gene over the 136 conditions sampled from the shoot tissue is higher than that over the remaining 136 conditions sampled from the root tissue. The average fold increase over all 382 hub genes is 45. In another experimental setting, gene expressions in response to light stimulus were compared with those under darkness conditions (contributed by Thomas Kretsch to AtGenExpress). We found that 97% of the 382 hub genes showed higher expression levels with the treatment of 4-hour continuous white light compared with the treatment of 4-hour continuous darkness (see Additional file 1 - Figure S1B); whereas only 54% of the 6206 genes in AGCN showed higher expression levels upon the light treatment. Thus, the 382 hub genes are highly expressed in shoots and up-regulated by light.

Based on the enrichment analysis of biological process and cellular component GO terms, pathway information and gene expression data, module 1 is likely to carry on the biological function of photosynthesis by coordinating more than 1000 genes' transcriptional activities. Based on this module-level annotation, many genes in module 1 with unknown functions would be hypothesized to be linked to photosynthesis [36].

### Module 4 - Cell Cycle

**Figure 10:**
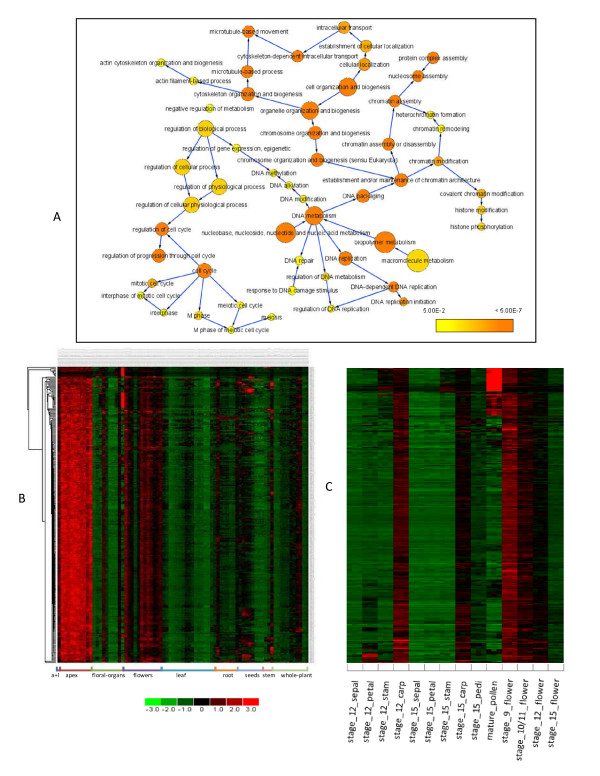
**Functional analysis of module 4**. (A) Significantly over-represented GO terms detected in module 4. (B) Co-expression patterns of 280 module genes over the 237 arrays which made up a gene expression map of *Arabidopsis *development [19]. In the heat map, each row represents a gene, and each column represents an array. Prior to hierarchical clustering, a gene's expression values over the 237 arrays were processed so that they had a zero mean and unit standard deviation. Arrays sampled from the same tissue were grouped together. 'a+l' represents the tissue that includes both shoot apex (vegetative) and young leaves. The heat map was generated using dChip software [68]. (C) A closer examination of the expression pattern of 280 module genes in different floral organs and whole flower tissues at different development stages. To generate the heat map, genes' expression values were extracted from the 280 × 237 data matrix, which were used to produce the heat map depicted in (B). Stage_XX represents a flower development stage, 'stam' represents stamen, 'carp' represents carpel, 'pedi' represents pedicel. For each experimental condition (*e.g*. stage_12_sepal), three replicates were measured.

Module 4 has 280 nodes, 5685 edges with a density of 0.15. Through consolidation, three major biological process GO terms were retrieved from the hierarchical relations of the GO terms that were over-represented in module 4 (Figure 10A): DNA metabolism (52 genes), cell cycle (33 genes), and cytoskeleton organization and biogenesis (26 genes). Altogether, these 111 genes account for 63% of the genes in the module with known biological processes.

Among the 33 cell cycle genes, 25 encode the cell cycle regulators that included 14 cyclins, 5 cyclin-dependent protein kinases (CDK), 2 members of the E2F transcription factors (E2F3 and DEL3), WEE1, MAD2, TSO2 and PCNA1. E2F transcription factors play important roles in pathways related to cell division, DNA repair, and differentiation[37]. WEE1, a protein kinase, controls cell cycle arrest by functioning as a DNA replication checkpoint [38]. MAD2 is a mitotic spindle checkpoint protein. TSO2 is a small subunit of ribonucleotide reductase (RNR) which is critical for cell cycle progression, DNA damage repair, and plant development [39]. *tso2 *mutants resulted in developmental defects, including callus-like floral organs and fasciated shoot apical meristems [39]. PCNA1 is a proliferating cell nuclear antigen, which is involved in DNA replication, DNA repair, chromatin remodeling, cell cycle regulation, and other functions [40]. Interestingly, *PCNA1 *is transcriptionally regulated by E2F [41].

The 52 DNA metabolism genes in module 4 included 23 genes involved in DNA replication, and 23 genes involved in chromatin assembly (mainly histone H2A/H2B/H3/H4) and modification (histone phosphorylation/methylation). The DNA metabolism process is apparently integrated with the cell cycle. Since cells orchestrated the coordinated progression through the cell cycle, the genes involved in DNA metabolism in module 4 are likely subject to the cell-cycle regulation. This is indeed the case. For example, *ORC1A, ORC1B*, *ORC3 *and *ORC4 *encode the subunits of Origin Recognition Complex which is involved in the initiation of DNA replication. The expressions of these four *ORC *genes are all regulated by E2F [42]. Another target of the E2F transcriptional factor, *FAS1*, encodes the chromatin assembly factor-1 (CAF-1) large subunit. Loss of *FAS1 *caused the inhibition of mitotic progression and triggered the endocycle program [43]. The regulation of *H4 *genes by another cell cycle regulator, TSO2, was demonstrated by the result that in a *tso2 *mutant, *H4*-expressing cells in flowers were dramatically increased compared with wild type, suggesting a prolonged S-phase in the mutant [39].

Among the 26 cytoskeleton organization and biogenesis genes, 19 encode kinesin motor proteins, 1 encodes *γ*-tubulin, and 4 encode actin-binding proteins. The kinesin motor proteins move along microtubules and play a role in mitosis by functioning in spindle formation, chromosome movement and cytokinesis [44]. Since multiple kinesin genes were detected in the co-expression module, their products might act cooperatively and play a role in the formation of mitotic microtubule arrays such as phragmoplast[44,45]. It was shown that several members of the kinesin protein family were probably regulated by CDK phosphorylation [44]. *γ*-Tubulin, is required for centrosomal and noncentrosomal microtubule nucleation and coordinates late mitotic events in *Arabidopsis *[46]. The functional role of actin cytoskeleton in the progression of cell cycle was well described in [47].

Since DNA metabolism, microtubule and actin organization and biogenesis carried by the genes in module 4 are all integrated with the cell cycle, the module is likely to carry on the function of cell cycle and cell proliferation. Since the cell cycle regulation is one of the keys to the control of plant development [48], we selected a data set from AtGenExpress which made up a gene expression map of *Arabidopsis *development [19]. The data set has 237 arrays which profiled many development stages, various tissues/organs, wild type and different mutants.

Figure 10B shows the expression profiles of the 280 genes in module 4 across the 237 arrays. Among different tissues, most of the 280 genes exhibited the highest expression levels in the shoot apex regardless of development stages, indicating that the module is active in the tissue enriched for cell dividing cells. The expression map of *Arabidopsis *development profiled the transcript abundance from the whole flower tissues from development stage 9 to 15. The map also included profiles from the four major floral organs (sepal, petal, stamen, carpel) at two development stages (12 and 15), pedicels at stage 15 and mature pollen. As shown in Figure 10B, most of the genes in module 4 were also highly expressed in flowers, another tissue enriched for cell dividing cells. However, they declined from relatively high expression to low expression (with respect to the average expression value of a gene over the 237 arrays) as the flower evolved from the development stage 9 to stage 15 (Figure 10C). With respect to the flower organs, most of the genes showed relatively high expression in carpels at stage 12 (Figure 10C). Such a distinct expression pattern implies that the cell-cycle specific module may play a role in the flower development. Indeed, three module genes, *ICU2*, *HTA8 *and *MET1*, function in the regulation of flower development. *ICU2 *encodes a catalytic subunit of the DNA polymerase a which is essential for the cell cycle by initiating DNA replication [49]. The *icu2-1 *mutant derepressed the expression of a number of regulatory genes including the ones involved in flowering time, floral meristem, and floral organ identity [49]. In the upstream, the expression of *ICU2 *is likely to be cell-cycle regulated based on the observation that its promoter contains an E2F binding site motif [49]. The second gene, *HTA8*, encodes a histone H2A protein. The gene knockdown experiment suggested the potential role of HTA8 in the regulation of flower development through the activation of *FLC*, a central floral repressor [50]. In human, a replication-dependent *H2A *gene was regulated by E2F in the early S phase of the cell cycle [51].  The third gene, *MET1*, encodes a cytosine methyltransferase. Demethylation of DNA brought about by a *MET1 *antisense caused early flowering in vernalization-responsive *Arabidopsis *[52]. *MET1 *was recently designated as a proliferation gene, and its expression is likely to be cell cycle dependent [53]. Thus, studying these three genes and probably other module members may help elucidate the mechanism underlying the linkage between the cell cycle regulation and the control of flower development.

In contrast to the shoot apex and flowers, nearly all of the genes in module 4 showed relatively low expression in leaves across all development stages (Figure 10B).

### Module 67 - Starch Metabolism

Compared with module 1 and 4, module 67 is a relatively small module with 10 nodes, 23 edges and a density of 0.51. Eight of ten genes in module 67 are involved in starch metabolism (p-value = 3.2E-19) based on their GO annotations. The other two genes are *AT3G46970 *and *AT2G28900*. *AT3G46970 *encodes a cytosolic alpha-glucan phosphorylase which was suggested to function as an enzyme of starch degradation [54]. *AT2G28900 *encodes an outer plastid envelope protein which was involved in the import of protochlorophyllide oxidoreductase A [55]. The expression of *AT2G28900 *gene was shown to be modulated by sucrose and responsive to a starchless mutant [56]. How this sugar sensing gene communicates with other starch metabolism genes remains to be elucidated.

### Comparison with GGM Network

In this study, we used standard Pcc to measure the degree of co-expression between two genes and connected them in the network if their Pcc is above a certain cutoff value. One concern with this approach is due to the transitive property of the standard correlation coefficient, which implies that if gene *A *and gene *B *are correlated with gene *C*, respectively, then *A *and *B *should be expected to correlate as well [5,13]. If this were true, then every gene with at least two neighbors in the AGCN would have a clustering coefficient close to 1. The plot displaying the relationship between the clustering coefficient (*C*_*k*_) and node degree (*k*) would approximately be a straight line of *C*_*k *_= 1. However, as shown in Figure 4B, *C*_*k *_showed a complex relationship with the node degree. Some sets of genes had relatively high clustering coefficients, suggesting the tight co-expression; while others had low clustering coefficients, indicating loose co-expression. As an example, among the 19 disconnected components each of which had exactly three nodes, 17 components had two edges connecting three genes whereas only 2 components had three edges, indicating only limited co-expression transitivity in AGCN. Nevertheless, we compared AGCN constructed in this study with the *Arabidopsis *gene network derived from a modified graphical Gaussian model (GGM), which used partial correlation as well as the standard Pearson correlation to select significantly correlated gene pairs [13].

In their study, a network of 18,625 edges connecting 6760 genes was obtained by using GGM [13]. The number of genes contained in the GGM-based network is close to that in AGCN constructed in this study. On the other hand, the average connectivity of a node in the GGM based network was 5.5, which is much smaller than that (165 links/node) in AGCN. Such a sparsely connected network, together with the number of highly connected genes being less than what would be expected according to the power-law distribution (Figure 3 in [13]), would be a challenge for computational methods to reliably detect a large number of modules [1]. Using the guide-gene approach, the authors retrieved subnetworks from the GGM based network. Each subnetwork included a seed gene and the genes that were within certain connecting steps from the seed gene. A functional module was then considered to be equivalent of the retrieved subnetwork itself. The disadvantage of this approach is that the retrieved subnetwork might be embedded within a larger module or it may include extra noisy genes. Nevertheless, the GO terms for many biochemical pathways (*e.g*. sulfate assimilation, cellular response to phosphate starvation, glycolipid metabolism, leucine catabolism, tryptophan metabolism, starch catabolism), cell wall metabolism, and cold response were significantly enriched in the subnetworks retrieved from the GGM-based gene network [13]. These GO terms were also significantly over-represented in the modules extracted from the AGCN (see the data files in our web site). However, with the guide-gene approach, the distinct major modules corresponding to photosynthesis (module 1 in AGCN), ribosome assembly and protein biosynthesis (module 3 in AGCN), and DNA metabolism and cell cycle (module 4 in AGCN), which are central to the plant growth and development, seemed to be absent from their report [13] probably because the correlations between many genes involved in these functions were determined to be insignificant by their partial correlation standard.

Since the gene modules of AGCN were detected by using the top-down approach, for a direct comparison, we also applied the same approach to the GGM network. Again, MCL algorithm was used to naturally partition the GGM network into modules. The functional coherence of obtained gene modules was assessed by using biological process GO terms (see our web site for the results). In general, clustering on the GGM network produced a larger number of modules but smaller module size compared with the clustering result of AGCN. For example, at 1.8 inflation value, MCL detected 1132 modules from the GGM network with the largest module only containing 45 nodes. With the same inflation value, MCL detected 527 modules from the AGCN with the largest modules containing 1381 nodes. Nine additional modules in AGCN also have more than 50 nodes. Although a large number of relatively small modules were detected in GGM network, interestingly, the distribution of module size fits very well to a power law distribution (Figure 4D). A power law distribution was also observed for module size of AGCN (Figure 4C).

Since a module is a densely connected subnetwork and the connections between modules are sparse, clustering on a network with internal modular structure should produce a large mass fraction close to one. However, it is not the case for GGM network (Figure 5B). For example, at 1.8 inflation value, clustering on GGM network captured 64% of the entire edge masses whereas AGCN captured 96% of edge masses. We also compared biological process GO term enrichment results between GGM and AGCN. Clustering on AGCN produced a slightly higher percentage of functionally coherent modules (Figure 7B). In the GGM network, the module with the most over-represented photosynthesis GO term (module 200, p-value = 2.60E-16) has 9 genes, the cell-cycle module (module 46, p-value = 6.77E-11) has 23 genes, and protein biosynthesis module (module 119, p-value = 2.08E-17) has 13 genes. In comparison, the corresponding gene functional modules in AGCN had much larger module sizes, and they were detected with the over-represented GO terms that were much more statistically significant (module 1, 3, 4 in Table 2).

To use GGM approach to construct genome-wide *Arabidopsis *gene network, a large number of samples are required. Since there are more than 20 thousand genes in *Arabidopsis*, a sample size comparable to the gene number is required to assess a full partial correlation for every gene pair [13]. In contrast, the standard Pcc approach does not require a large sample size. It can be used to analyze small data sets that are typically seen in focused microarray experiments. As a proof of concept, we applied our network-based approach to a relatively small data set. The data set profiles global gene expression in shoot in response to continuous cold stress (4°C). The gene expression was measured at 7 time points (0, 0.5, 1, 3, 6, 12 and 24 h) [18]. For each time point two samples serving as replicates were analyzed. Thus, the data set consists of a total of 14 samples. Of the 22746 *Arabidopsis *probe sets on the ATH1 chip, 4915 (22%) were selected as the genes that showed significant changes over the 14 samples (see Methods). Pcc value for each pair of the 4915 genes was calculated and sorted. The top 0.39% of gene pairs with respect to their Pcc values were used to construct the cold induced gene co-expression network. The same percentage of gene pairs were retained in the 1094-array AGCN. The resulting cold induced gene network has 1700 nodes, 46671 edges, and a network density of 0.0323.

Following the construction of cold induced gene network, MCL algorithm was used to partition the network into modules. 84 modules with at least three genes were obtained at 1.8 inflation value. Of these modules, 18 (21.4%) had biological process GO terms that were significantly over-represented (see our web site for the results). Among the enriched GO terms, 'response to auxin stimulus' is the most over-represented (module 31, p-value = 4.13E-8). It has been reported that auxin responsive genes were regulated by cold stress which may contribute to the alteration of plant growth to coordinate with cold [57]. 'Cellular carbohydrate metabolism' is the second most over-represented biological process GO term, and it was identified in module 1 (p-value = 9.31E-08). The module includes genes involved in starch, sucrose, trehalose and glucose metabolism. Starch and sugars play an important role in the biochemical adaption of plant to cold [58-60]. Other GO terms for biological processes involved in transcription regulation, cellular defense, stress response, and signal transduction are also significantly over-represented. Taken together, these results can help us better understand the molecular mechanisms of plant cold responses. It demonstrates that our network-based approach can be applied to analyze small data sets and identify gene modules important to specific biological questions.

## Discussion

In this study, we used a top-down approach (or non-targeted approach) to naturally partition the genome-wide *Arabidopsis *gene co-expression network into gene modules based on the topological property of the network. We used an efficient graph clustering algorithm to identify modules from the AGCN. Compared with the traditional clustering analysis such as hierarchical clustering and k-means clustering, the network approach provides additional structural information regarding the connectivity of genes [61]. Genes belonging to the same module are not only highly correlated at the expression level but also densely connected to each other. Thus, compared with a cluster obtained from traditional clustering methods, a module detected from the network is a more tightly controlled structure which would be more biologically meaningful and resilient to data noise [4,8,61].

The constructed AGCN and its extracted modules showed the following properties: (1) The distribution of the node degree fits to a power law distribution (Figure 4A). Such distribution was also observed in the human gene co-expression network and conserved gene co-expression network derived from the human, fly, worm and yeast comparisons [5,9]. (2) The hub genes in the AGCN were densely connected to each other as shown by the clique structure formed by the 382 hub genes. Although it contradicts with a commonly held view that hub nodes tend not to link to each other, it has been recently reported that hub-hub interactions were not suppressed in a multi-validated high-confidence protein interaction network for yeast [21]. In addition it was shown that nodes in gene co-expression networks tended to connect with the ones with similar degrees while the connections between highly and lowly connected nodes were suppressed [10].  Here, we further argue that the preferential hub-hub connections would be necessary for the formation of modular structure in gene co-expression networks. The hub genes which are densely connected to each other would be self-contained in a single module. In our case, the 382 hub genes were embedded in module 1. In contrast, for the random networks, which had the same distribution of node degrees as AGCN but in which the connection between a hub and a low degree gene would not be expected to be suppressed, our results clearly indicate the lack of modular structure. (3) The average clustering coefficient of AGCN was increased by more than one fold compared with the random networks, supporting the modular structure in AGCN. (4) Similar to the node degree distribution, the distribution of the module size also follows a power law distribution (Figure 4C). Interestingly, the distribution of the sizes of 400 complexes extracted from the yeast protein interaction network also displayed the power law distribution [23]. The biological implication of the power law distribution of the module size remains to be further investigated.

The approach used in this study, constructing a gene co-expression network and naturally partitioning the network into modules, provided a systems-level understanding of the gene modules that coordinate multiple biological processes to carry out specific biological functions. Plants convert light energy to chemical energy through photosynthesis. Our results suggested that photosynthesis module in *Arabidopsis *involves a very large number (> 1000) of genes which participate in photosynthesis and related biological processes. The related biological processes encompassed protein biosynthesis, electron transport, cofactor metabolism, chloroplast organization and biogenesis, pigment metabolism, and vitamin metabolism. The GO terms for these biological processes were all significantly over-represented, suggesting the important roles of these biological processes in photosynthesis. Nevertheless, other biological processes which were not significantly over-represented might also play a role in photosynthesis. Different from most animals, plants develop continuously with new organs being developed throughout the lifetime of the plant [19]. The cell cycle regulation is one of the keys to the control of plant development. The cell cycle module detected from AGCN orchestrated the coordinated expression of hundreds of genes participating in cell cycle, DNA metabolism, and cytoskeleton organization and biogenesis. Interestingly, a human cell cycle module, which was obtained from an integrated analysis of ~2000 cancer arrays, gene ontology and pathways, contained approximately the same number of genes (263) and similar gene function compositions [62]. In this report, we studied the gene co-expression network and functional modules for *Arabidopsis*. The same approach should be applicable to other model organisms.

Genes in the same module are co-expressed across diverse conditions, suggesting the potential underlying co-regulation mechanism. The list of gene modules obtained in this study would provide a useful tool for the regulation investigation. One approach to linking co-expression to co-regulation is to examine the putative transcription factor binding sites (TFBS) in the promoters of the co-expressed genes [14].

## Conclusion

In this study, we used a network-based approach to identify gene functional modules from large microarray data sets of *Arabidopsis thaliana*. The study reveals new insight into the topological properties of biological networks. The preferential hub-hub connections might be necessary for the formation of modular structure in gene co-expression networks. The study also reveals new insight into the organization of gene functional modules.

## Methods

### Normalization and Pcc calculation

The 1094 arrays from AtGenExpress were normalized using the justMAS function in the simpleaffy package (version 2.8.0) [63] downloaded from Bioconductor with the target value set to 500. After the normalization, genes satisfying the following two conditions were selected for the further analysis: (1) the ratio between standard deviation and mean of a gene's expression values over the 1094 arrays is greater than 0.5; (2) the difference between a gene's maximal expression value and minimal value among the 1094 arrays is greater than 32. After the filtration, the remained genes' expression values were *log*_10 _transformed. During the transformation, if a gene's expression value was less than 1, its *log*_10 _transformed value was converted to 0 instead of a negative number. The Pearson correlation coefficient between two genes over the 1094 arrays was calculated using their *log*_10 _transformed values.

We also applied our network-based approach to a small data set which consists of 14 samples. The data set profiles gene expression in response to cold stress which is part of AtGenExpress [18]. We used the above two criteria to select genes that showed significant changes across the 14 samples. After the filtration, the remained genes' expression values were *log*_10 _transformed, and the Pearson correlation coefficients for gene pairs over the 14 samples were calculated.

### Network Analysis

Node degree indicates the number of links connected by a node. Network density is defined as a ratio of the observed number of edges to all possible edges among the network nodes. The clustering coefficient (*C*_*n*_) of a given node *n *was calculated as the following. Assuming the node *n *has *k *(*k *≥ 2) directly connected neighbors, then



where *e(k) *is the observed number of edges among the *k *neighbors. <*C*_*n*_> represents the average clustering coefficient of the network over all nodes which have at least two neighbors. The clustering coefficient (*C*_*k*_) with respect to the node degree *k *is the average over all nodes each of which has exact *k *neighbors.

Mass fraction, area fraction and efficiency were used to quantify the overall quality of the network clustering. The mass fraction is defined as the following. Let *e *be an edge of the network. The clustering captures *e *if the two nodes connected by *e *belong to the same module (cluster). Now the mass fraction is the ratio between the joint weights (Pearson correlation coefficients) of all captured edges over all modules and the joint weights of all edges in the network [17]. The area fraction, *AF*, is calculated as the following



where *M *is the number of modules extracted from the network, *N*_*i *_is the number of nodes contained in the *i*^*th *^module, and *N *is the number of nodes in the network. A low area fraction indicates a fine-grained clustering whereas a high area fraction indicates a coarse clustering [17]. The efficiency aims to balance between the objective to obtain a high mass fraction and the objective to keep the area fraction low. The formal definition of efficiency can be found in [64].

The random network, which assumed the same node degree distribution as the AGCN, was generated using the randomNodeGraph function in the R package Graph (version 1.15.6).

### GO/Pathway Term Enrichment Analysis

The GO terms for *Arabidopsis *loci were downloaded from . The GO terms were then assigned to array probe sets based on the correspondence between the probe sets and loci obtained from The Arabidopsis Information Resource (TAIR) [26]. GO term enrichment analysis was carried out by using BiNGO 2.0 [65]. Bonferroni Family-Wise Error Rate (FWER) correction was used to control the false positive rate. If a GO term in a module showed a FWER corrected p value less than 0.05 in comparing with the AGCN, which comprised of 6206 probe sets, under a hypergeometric distribution, then the GO term was determined to be significantly enriched in this module.

For some module, more than 50 biological process GO terms were significantly over-represented. To simplify our functional annotation of the module, these GO terms were consolidated to obtain a small set of representative major GO terms. Firstly, the GO terms that were too general (*e.g*. macromolecule metabolism) were manually discarded. Secondly, based on parent-child relationships depicted in a hierarchical graph of over-represented GO terms (*e.g*. Figure 10A), the GO terms that were at the top level were manually retrieved from the graph to represent the GO terms that were at lower levels. Thirdly, genes annotated to the GO terms that were retrieved in step 2 were inspected so that each gene is only associated with one GO term. Thus, the retrieved major GO terms are associated with non-overlapping gene sets.

The pathway information for *Arabidopsis *genes was obtained from AraCyc 4.0 [66]. The criteria to detect the significantly enriched pathway terms in a module are same as those to detect GO terms.

## Availability

The results for the AGCN, three random networks and GGM network can be accessed at the url 

## Abbreviations

AGCN: *Arabidopsis *gene co-expression network

## Authors' contributions

LM designed the project, conducted the project and wrote the manuscript. JVH developed some data analysis tools. SD modified the manuscript. JD oversaw the project and modified the manuscript. All authors read and approved the final manuscript.

## Supplementary Material

Additional File 1 **Supplemental Material**. the document includes (1) F igure S1 showing the expression patterns of the 382 hub genes in shoot and root tissuse, in response to light stimulus; (2) Table S1 listing 63 genes involved in protein biosynthesis in module 1 whose products might be located in chloroplast; (3) Table S2 listing over-represented pathway terms in module 1. Click here for file
