# Supplementary material for: Arabidopsis gene co-expression network and its functional modules
Source: BMC Bioinformatics. 2009 Oct 21;10:346. doi: 10.1186/1471-2105-10-346 (PMC2772859; doi:10.1186/1471-2105-10-346)
Supplement: Additional File 1 — Supplemental Material. the document includes (1) F igure S1 showing the expression patterns of the 382 hub genes in shoot and root tissuse, in response to light stimulus; (2) Table S1 listing 63 genes involved in protein biosynthesis in module 1 whose products might be located in chloroplast; (3) Table S2 listing over-represented pathway terms in module 1. [file 1471-2105-10-346-S1.PDF]

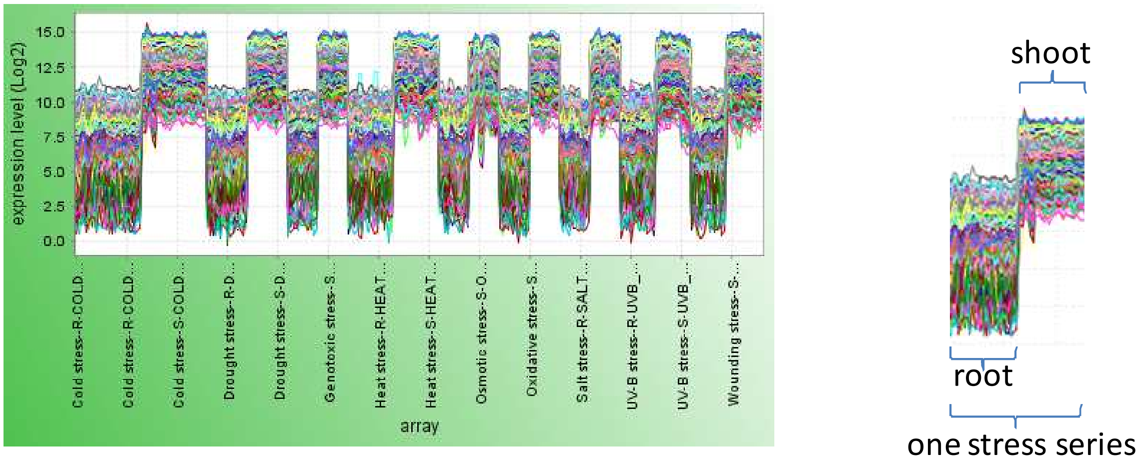

A

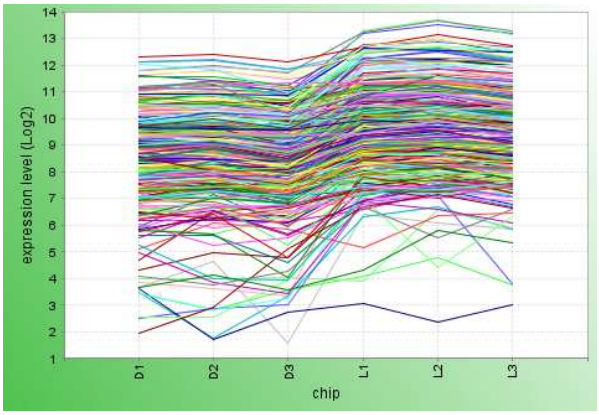

B

Supplementary Figure 1. Expression patterns of the 382 hub genes. (A) The expression of the 382 hub genes across the 272 arrays. These 272 arrays consisted of nine stress series which were arranged in the following order: cold, drought, genotoxic, heat, osmotic, oxidative, salt, UV-B, and wounding stress. For each stress, the gene expression in root was compared with the expression in shoot. (B) 382 hub genes response to light stimulus. D1, D2, and D3 are the three replicates for the treatment of 4-hour continuous darkness, whereas L1, L2, and L3 are the three replicates for the treatment of 4-hour continuous white light.

Supplementary Table 1. 63 genes involved in protein biosynthesis in module 1 whose products might be located in chloroplast

Array Element	Locus Identifier	Annotation
249331_at	*AT5G40950*	50S ribosomal protein L27, chloroplast, putative (RPL27)
261119_at	*AT1G75350*	EMB2184 (EMBRYO DEFECTIVE 2184); structural constituent of ribosome
265247_at	*AT2G43030*	ribosomal protein L3 family protein
245049_at	*ATCG00050*	Homologous to the bacterial ribosomal protein S16
266648_at	*AT2G25840*	OVA4 (OVULE ABORTION 4); ATP binding / aminoacyl-tRNA ligase
258674_at	*AT3G08740*	elongation factor P (EF-P) family protein
250146_at	*AT5G14660*	PDF1B (PEPTIDE DEFORMYLASE 1B); peptide deformylase
257932_at	*AT3G17040*	HCF107 (HIGH CHLOROPHYLL FLUORESCENT 107); binding
262172_at	*AT1G74970*	RPS9 (RIBOSOMAL PROTEIN S9); structural constituent of ribosome
251883_at	*AT3G54210*	ribosomal protein L17 family protein
249742_at	*AT5G24490*	30S ribosomal protein, putative
262029_at	*AT1G35680*	50S ribosomal protein L21, chloroplast / CL21 (RPL21)
244988_s_at	*ATCG00840; ATCG01300*	[ATCG00840, One of two chloroplast genes that encode chloroplast ribosomal protein L23, a constituent of the large subunit of the ribosomal complex];[ATCG01300, One of two chloroplast genes that encode chloroplast ribosomal protein L23, a constituent of the large subunit of the ribosomal complex]
256855_at	*AT3G15190*	chloroplast 30S ribosomal protein S20, putative
245852_at	*AT5G13510*	ribosomal protein L10 family protein
250190_at	*AT5G14320*	30S ribosomal protein S13, chloroplast (CS13)
254910_at	*AT4G11175*	translation initiation factor IF-1, chloroplast, putative
261954_at	*AT1G64510*	ribosomal protein S6 family protein
245009_at	*ATCG00380*	Chloroplast encoded ribosomal protein S4
251120_at	*AT3G63490*	ribosomal protein L1 family protein
260898_at	*AT1G29070*	ribosomal protein L34 family protein
244986_at	*ATCG00820*	Encodes a 6.8-kDa protein of the small ribosomal subunit.
261078_at	*AT1G07320*	RPL4 (ribosomal protein L4); structural constituent of ribosome
249554_at	*AT5G38290*	peptidyl-tRNA hydrolase family protein
262283_at	*AT1G68590*	plastid-specific 30S ribosomal protein 3, putative / PSRP-3, putative
266570_at	*AT2G24090*	ribosomal protein L35 family protein
250247_at	*AT5G13720*	structural constituent of ribosome
248798_at	*AT5G47190*	ribosomal protein L19 family protein
267435_at	*AT2G33800*	ribosomal protein S5 family protein
248634_at	*AT5G49030*	OVA2 (OVULE ABORTION 2); ATP binding / aminoacyl-tRNA ligase
253597_at	*AT4G30690*	translation initiation factor 3 (IF-3) family protein
264575_at	*AT1G05190*	EMB2394 (EMBRYO DEFECTIVE 2394); structural constituent of ribosome

246339_at	*AT3G44890*	RPL9 (ribosomal protein L9); structural constituent of ribosome
255850_at	*AT2G33450*	50S ribosomal protein L28, chloroplast (CL28)
251573_at	*AT3G58140*	phenylalanyl-tRNA synthetase class IIc family protein
253758_at	*AT4G29060*	EMB2726 (EMBRYO DEFECTIVE 2726); translation elongation factor
245357_at	*AT4G17560*	ribosomal protein L19 family protein
255328_at	*AT4G04350*	EMB2369 (EMBRYO DEFECTIVE 2369); ATP binding / aminoacyl-tRNA ligase
262235_at	*AT1G48350*	ribosomal protein L18 family protein
247201_at	*AT5G65220*	ribosomal protein L29 family protein
260165_at	*AT1G79850*	RPS17 (ribosomal protein S17); structural constituent of ribosome
244960_at	*ATCG01020*	encodes a chloroplast ribosomal protein L32, a constituent of the large subunit of the ribosomal complex
257190_at	*AT3G13120*	30S ribosomal protein S10, chloroplast, putative
253384_at	*AT4G32915*	glutamyl-tRNA(Gln) amidotransferase
262483_at	*AT1G17220*	translation initiation factor IF-2, chloroplast, putative
255623_at	*AT4G01310*	ribosomal protein L5 family protein
244968_at	*ATCG00640*	encodes a chloroplast ribosomal protein L33, a constituent of the large subunit of the ribosomal complex
266478_at	*AT2G31170*	tRNA synthetase class I (C) family protein
251172_at	*AT3G63190*	ribosome recycling factor, chloroplast, putative / ribosome releasing factor, chloroplast, putative
257225_s_at	*AT3G27850; AT3G27830*	[AT3G27850, RPL12-C (RIBOSOMAL PROTEIN L12-C); structural constituent of ribosome];[AT3G27830, RPL12-A (RIBOSOMAL PROTEIN L12-A); structural constituent of ribosome]
254480_at	*AT4G20360*	AtRABE1b/AtRab8D (Arabidopsis Rab GTPase homolog E1b); translation elongation factor
248174_at	*AT5G54600*	50S ribosomal protein L24, chloroplast (CL24)
253295_at	*AT4G33760*	tRNA synthetase class II (D, K and N) family protein
245413_at	*AT4G17300*	NS1 (OVULE ABORTION 8)
249691_at	*AT5G36170*	HCF109 (HIGH CHLOROPHYLL FLUORESCENT 109); translation release factor
246509_at	*AT5G16715*	EMB2247 (EMBRYO DEFECTIVE 2247); ATP binding / aminoacyl-tRNA ligase
252404_at	*AT3G48110*	EDD1 (EMBRYO-DEFECTIVE-DEVELOPMENT 1)
256728_at	*AT3G25660*	glutamyl-tRNA(Gln) amidotransferase, putative
244969_at	*ATCG00650*	chloroplast-encoded ribosomal protein S18
244996_at	*ATCG00160*	Chloroplast ribosomal protein S2
263131_at	*AT1G78630*	EMB1473 (EMBRYO DEFECTIVE 1473); structural constituent of ribosome
246673_at	*AT5G30510*	RPS1 (ribosomal protein S1); RNA binding
266575_at	*AT2G24060*	translation initiation factor 3 (IF-3) family protein

Supplementary table 2. Over-represented pathway terms in module 1.

Pathway	# genes in pathway[1]	P value[2]
photosynthesis, light reaction	27/27	4.69E-15
photosynthesis, dark reaction	21/25	3.26E-08
chlorophyllide <i>a</i> biosynthesis	13/14	1.83E-06
carotenoid biosynthesis	9/10	1.68E-04

1. The two values separated by '/' refer to the number of genes annotated to the over-represented pathway in the module and the number of genes annotated to the same pathway in the network.

2. The p value indicated the probability that the module contains equal or larger number of genes associated with the pathway term under a hypergeometric distribution.
